# Implementing predictive tools in surgery: A narrative review in the context of orthopaedic surgery

**DOI:** 10.1111/ans.18044

**Published:** 2022-09-15

**Authors:** Yuxuan Zhou, Daniel Gould, Peter Choong, Michelle Dowsey, Chris Schilling

**Affiliations:** ^1^ Department of Surgery The University of Melbourne Melbourne Victoria Australia

**Keywords:** artificial intelligence, clinical decision aids, clinical predictive tools, diagnostic tools, external validation, implementation, machine learning, orthopaedic surgery, prognostic tools, translation

## Abstract

Clinical predictive tools are a topic gaining interest. Many tools are developed each year to predict various outcomes in medicine and surgery. However, the proportion of predictive tools that are implemented in clinical practice is small in comparison to the total number of tools developed. This narrative review presents key principles to guide the translation of predictive tools from academic bodies of work into useful tools that complement clinical practice. Our review identified the following principles: (1) identifying a clinical gap, (2) selecting a target user or population, (3) optimizing predictive tool performance, (4) externally validating predictive tools, (5) marketing and disseminating the tool, (6) navigating the challenges of integrating a tool into existing healthcare systems, and (7) developing an ongoing monitoring and evaluation strategy. Although the review focuses on examples in orthopaedic surgery, the principles can be applied to other disciplines in medicine and surgery.

## Graphical abstract

Clinical predictive tools are a topic gaining interest. Of all predictive tools developed, only a small proportion are implemented into clinical practice. In this narrative review, we discuss the key principles that guide the translation of predictive tools into clinical practice. We focus on examples in orthopaedic surgery. However, the principles discussed can be applied to other aspects of medicine and surgery.

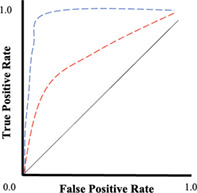

## Introduction

Predictive tools are becoming more widespread in clinical practice.^1^, ^2^, ^3^, ^4^, ^5^, ^6^ Commonly, these tools are used to prognosticate (prognostic tools) or diagnose (diagnostic tools) medical conditions or events, although the use of predictive tools can extend beyond these roles. They are often developed from large datasets, with thousands or even millions of data points processed.^7^, ^8^ Traditional statistical approaches and/or machine learning (ML) algorithms are used on these datasets to predict future outcomes. The development process often incorporates concepts around ‘big data analysis’, which uses computer programmes to undertake iterative calculations that otherwise would be impossible to perform manually.^9^

Despite the increasing popularity of predictive tool development, the concept of using predictive tools to assist clinical decision‐making has been practiced since ancient Babylonian times.^10^ During those times, predictive tools took the form of diagnostic handbooks to present logical rules combining observed symptoms with diagnosis and prognosis. In the modern era, predictive tools often take advantage of digital technology in the form of online calculators or mobile applications, although the principles underlying their predictions have remained largely unchanged over time.^11^, ^12^, ^13^ Learnings from past experiences, housed in observational data, remain the mainstay of predictive modelling. In addition, platforms such as *MDCalc* act as an easy‐to‐access online repository for evidence‐based clinical decision support (CDS) tools, of which predictive tools are a subgroup.^14^

Predictive tools are of optimal benefit when they are used in a complementary capacity with the clinician.^15^, ^16^ Modern predictive tools harness the summated experience of thousands of previous events to generate predictions. This combined experience often far exceeds what is possible for a single clinician to accumulate in their entire career. Predictive tools have the potential to minimize conscious (and subconscious) biases inherent to the human clinical decision‐making process.^17^ The flow‐on effect of bias minimisation is the promotion of equitable health provision through the standardization of care.^18^ Clinicians can use predictive tools to aid in the diagnosis or management of patients based on evidence and risk calculations.

Although the benefits of predictive tools are plentiful, there are also pitfalls that must be considered when using them. Most commonly, predictive tools can mistakenly be used on patients or populations for whom they were not intended.^19^ Similarly, problems arise when predictive tools are used in a context for which they were not designed. An example illustrating these concerns can be demonstrated with the Mirel's score for prophylactic fixation.^20^ This tool was originally designed to predict the risk of pathological fracture in metastatic lesions prior to irradiation. However, when used on patients with benign lesions or patients who have additional functional risks such as falls, the predictive outcome may not be accurate. As a result, additional interventions such as unnecessary ionizing scans or even surgery may be performed causing harm to the patient and adding significant costs to the health system.^21^ This highlights the need for clinical acumen to be used alongside predictive tools.

In clinical practice, many predictive tools are already used routinely. For example, the FRAX tool used to predict osteoporotic fracture has been widely used since 2010.^22^ Other CDS tools such as the TLICS score used to guide operative or non‐operative surgery in spinal trauma has been adopted widely.^23^, ^24^ Similar tools have been used in almost every medical specialty with varying degrees of success.^25^, ^26^, ^27^, ^28^, ^29^, ^30^, ^31^, ^32^ However, for every successful predictive tool used in clinical practice, there are many more tools that have been developed but are never utilized in clinical practice.^33^

Therefore, this review aims to explore the factors needed to implement predictive tools into clinical practice. Specifically, we discuss key principles to successfully transition predictive tools from academic bodies of work into effective tools that can be used in routine clinical practice.

## Narrative synthesis

A narrative synthesis method was used for this review rather than systematic review with meta‐analysis. The rationale for this methodology was because; (1) narrative reviews allowed for a broader and less restrictive scope of content, (2) there were insufficient articles in the literature with common aims to perform a systematic review with meta‐analysis, and (3) the narrative review provided a better opportunity to present both factual evidence and author interpretation of the evidence.^34^ For a topic such as implementing predictive tools in clinical practice, there is a range of perspectives for how this process could be optimized, which is summarized in this narrative review.

A general search of the literature was first performed using the keywords (OR) ‘prognostic tool’, ‘diagnostic tool’, ‘predictive model’, ‘predictive tool’, ‘linear regression’, ‘logistical regression’, ‘multivar* regression’, ‘machine learning’, and ‘artificial intelligence’. Subsequently, the keywords were linked with refined terms (AND): ‘translation’, ‘implementation’, ‘integration’, and ‘adoption’ to ‘clinical practice’. The search was performed on MEDLINE (Ovid), Embase, and Google Scholar. References from articles found in the literature search and citations of the articles were also reviewed. Only articles written in English were included in the synthesis. No time restrictions were imposed on article publication date. Articles were screened based on title and abstract relevance by two authors (YZ & DG), then reviewed in full text. Commentary from experts in the field of predictive modelling, both clinical and academic, were used to complement the findings from the literature search. These included opinion pieces, editorials, lectures, and interviews. Common concepts were then synthesized using reflective thematic analysis.^35^ Final inclusion into the narrative review was based on two tiers of consideration. The first consideration being themes relevant to the aims of the review and the second being themes that followed the chronology of predictive tool implementation. The results were reported as a narrative review aligning with recommendations from the Scale for the Assessment of Narrative Review Articles (SANRA) guidelines.^36^

Based on thematic analysis of the literature search, seven key areas of consideration for predictive tool development were identified and will be discussed further:

- Identifying an appropriate clinical gap
- Selecting a target user and population
- Optimizing predictive performance
- External validation
- Marketing and dissemination
- Integration into existing healthcare systems
- Continuous monitoring and evaluation

**Fig. 1:**
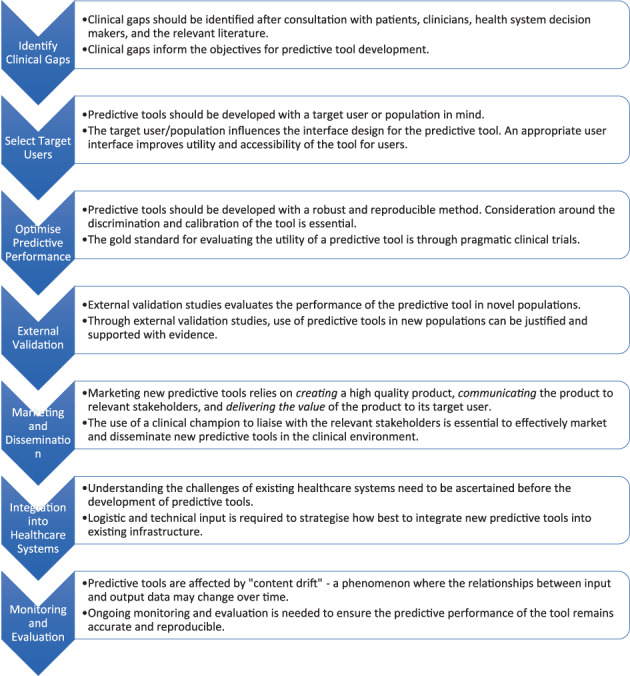
The implementation principles in schematic form to transition new predictive tools into a clinical environment.

These seven principles form the foundation of what is required to implement predictive tools successfully (Fig. 1). They are in chronological order of tasks that need to be performed. Although each individual area is important, consideration of all seven areas as a collective is paramount for transitioning predictive tools into clinical practice.

## Identifying clinical gaps

A new intervention in clinical practice should address a clinical gap. This could arise from existing interventions that require improvement or from novel areas in medicine where interventions do not currently exist. To identify where a gap exists in clinical practice, consultation from four sources should be considered—the patient, the clinician, the health system, and the body of relevant literature.^37^, ^38^, ^39^ For the patient, their perspective represents the recipients of healthcare services. The end goal here is to improve the healthcare experience for the patient. In contrast, the perspective of the clinician provides insight from those who deliver healthcare at the coal face. Clinicians can provide an anecdotal experience of where the problems lie in healthcare provision. From this, clinical gaps can be identified for new interventions to improve parameters such as diagnostic accuracy and clinical efficiency. Beyond individual interactions, the healthcare system as an entity needs to be considered and consulted. This includes the perspectives of hospital management, health economists, and politicians who make decisions about resource allocation. Although specific clinical gaps may not be identified through this source alone, an overarching view of where interventions may meet the highest need can be determined.^40^ Finally, the competing interests of all parties need to be reconciled with the relevant evidence. This should represent an unbiased viewpoint of where clinical gaps have been identified systematically and how best to address these gaps using new interventions.

In the context of predictive tools, a significant clinical gap should be identified to produce a clear objective for its development. Clinical gaps can be determined based on local needs (e.g., clinical audit recommendations) or national needs (e.g., from national health prioritization task forces).^41^ In addition, the predictive outcome of the tool should align with the clinical gap(s) that needs to be addressed.

## Selecting target users and populations

Predictive tools should be developed with the target users and populations in mind. This may influence which predictors and outcomes are most relevant for its user. For example, a predictive tool incorporating blood test results may not be appropriate if intended for patients to use *before* seeing a doctor. Similarly, if a predictive tool is intended for use by clinicians during a busy clinic, then consideration may be required for time required to use the tool.

Optimal user interface (UI) design of predictive tools requires knowledge of the target audience.^42^, ^43^, ^44^ If using an example of total knee arthroplasty (TKA), patients are often older and from a generation where digital technologies were not so commonplace.^45^ Therefore, UI design for predictive tools targeting this patient population should be age‐appropriate and easily accessible. Complex apps requiring special software may not be suitable for target users who may not be familiar with advanced computer programs. In conjunction with an appropriate UI, education surrounding tool use may complement its accessibility.^46^ Using the TKA target population, additional education interventions on computer and internet access may be required to optimize the use of predictive tools in these patients.

Having a target audience in mind before the development of a predictive tool establishes boundaries for how the tool will function. User uptake of predictive tools may improve if they are designed with the needs of their target user taken into consideration.^47^

## Optimizing predictive performance

One of the key barriers preventing the implementation of novel predictive tools in routine practice is clinician distrust.^48^ Some of the commonly used predictive tools in clinical practice lack evidence to support their use in specific populations or clinical scenarios.^49^ This lack of evidence stems from both a paucity of clinical trial data using predictive tools and the absence of standardized methods for evaluating predictive tools in a clinical context. In an age of scepticism, predictive tools face the same challenges as many other interventions in clinical medicine. Information is freely available and accessible, which can both support and condemn the use of new interventions. Therefore, predictive tools need to be able to navigate the scrutiny of public opinion.

**Fig. 2:**
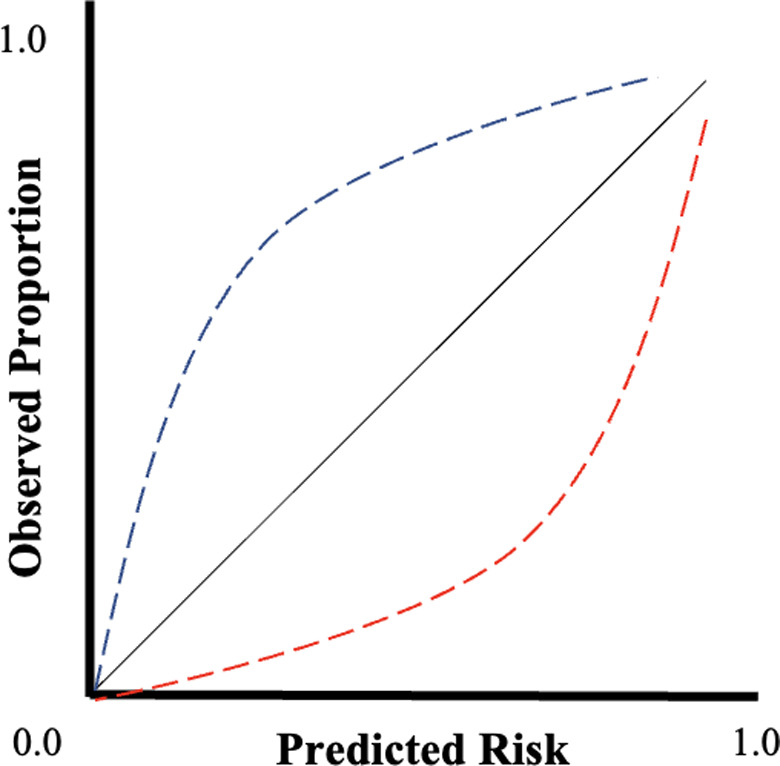
Calibration plot illustrating the estimation of risks. The diagonal black line demonstrates perfect estimation (intercept = 0, slope = 1); the blue dashed line demonstrates underestimated risks (intercept = 1.00, slope = 1); and the red dashed line demonstrates overestimated risks (intercept = −1.25, slope = 1).

**Fig. 3:**
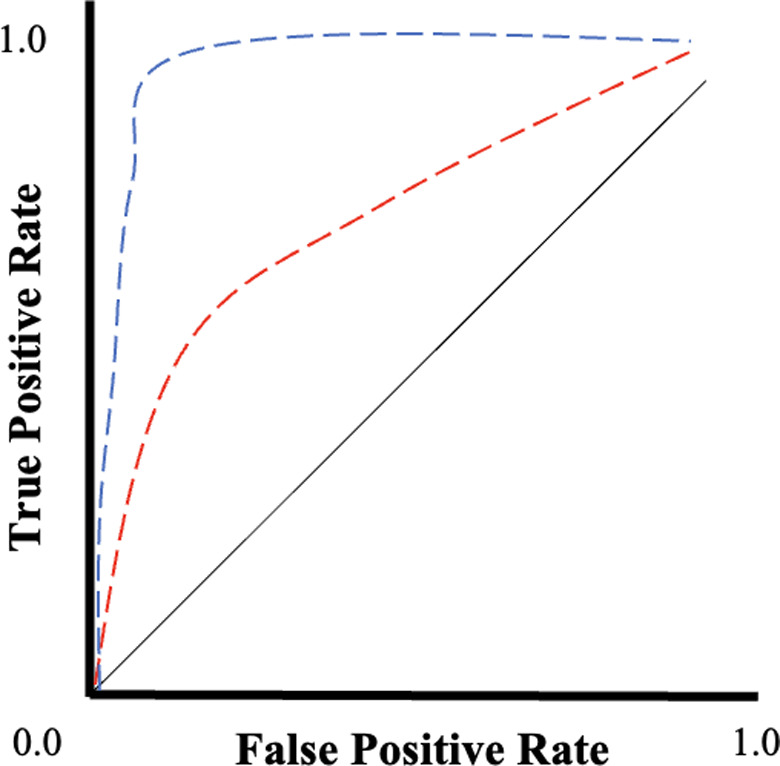
Receiver operator curve illustrating how well a predictive model can classify predictions. The diagonal black line represents a random classifier; the red dashed line represents a good classifier; and the blue dashed line represents the best classifier in this series.

To do this, predictive tools should be supported by a solid foundation of evidence.^50^ This process begins at the development stage. Predictive modelling techniques should be robust and reflect a high degree of statistical and clinical accuracy. Reporting of predictive tool development should follow standardized and transparent guidelines. The Transparent reporting of a multivariate prediction model for individual prognosis or diagnosis (TRIPOD) statement aims to provide an open access guideline for the standardized reporting of predictive tool development.^51^ In addition, common metrics such as *R* ^2^ values, root mean squared error (RMSE), sensitivity and specificity should be reported openly when relevant. Additional concepts should be considered when developing a new predictive tool. These are the calibration and discrimination attributed to the tool. Calibration measures the agreement between predicted risks and the proportion of patients with the outcome. It is usually split into deciles, with 0–1 on the *x*‐axis representing predicted probabilities and 0–1 on the *y*‐axis representing the proportion of patients with the outcome of interest (Fig. 2). The perfectly calibrated predictive model is on the diagonal line, indicating that the proportion of patients with a predictive risk is equal to the proportion of patients who have the outcome. In comparison, discrimination in a predictive model represents the proportion of cases in which the model distinguishes between patients who get the outcome and those who do not. This is often plotted as a receiver operator curve (ROC), which illustrates how well a model can classify its predictions (Fig. 3). The ROC plots the true positive rate against the false positive rate (or alternatively sensitivity against 1‐specificity). To quantify how well a model can classify its predictions, the AUC is calculated from the ROC. In other words, an AUC of 0.7 discriminates between patients with the outcome and those without the outcome 70% of the time.

Finally, the gold standard for supportive evidence of predictive tool use is clinical trials.^52^ Ideally, these should be prospective, multicentred, blinded, cluster randomized controlled trials evaluating the predicted outcome of a specific tool. Although costly and logistically challenging to organize, evidence gathered from clinical trials provides a pragmatic understanding of how predictive tools would function in routine clinical practice.^53^ The limitation for clinical trial use is that diagnostic tools may not be appropriate as a study intervention due to ethical considerations. For diagnostic tools, the next best alternative to provide evidence of its clinical performance is through comparator studies between diagnostic tools and clinicians accumen.^54^

## External validation

Most predictive tools use a large dataset that is arbitrarily split into ‘training’ and ‘testing’ samples. The training sample (usually 75% of the dataset) is used to develop a predictive model, and the testing sample is used to evaluate the fit of the predictive model. Although this technique is a method of validating the predictive model, it is considered a form of internal validation, as both the training and testing samples are derived from a single dataset. Cross‐validation and bootstrapping can be used to generate different permutations of training and testing data. However, this also remains limited as an internal validation technique.

Further evidence for predictive tools must then come from external validation studies.^55^ This involves the evaluation of a developed predictive tool on novel populations.^19^, ^56^ Through external validation, confounding factors that may bias the results between different populations can be addressed. These confounding factors can include differences in socioeconomic status, healthcare resources or personal characteristics such as body mass index (BMI). External validation on diverse populations can expand the scope for predictive tool use. This is particularly important for predictive tools intended to be used in conditions that are widely prevalent across many populations.^57^ Examples of these conditions include predictive tools for appendicitis, myocardial infarctions, and arthritis.

## Marketing and dissemination

Hospitals and other healthcare institutions are continuously presented with multiple competing interventions and ideas. Therefore, a marketing strategy for new predictive tools is vital for their successful implementation in clinical practice. To develop an effective strategy, it is necessary to first understand how marketing supports the implementation of new interventions in healthcare. Clarke *et al*. summarized marketing as ‘the customer‐focused philosophy with the acronym CCDV; the aim of marketing is to *create, communicate, and deliver value*’.^58^

The *creation* aspect of marketing has already been addressed in this review with emphasis on the technical considerations for developing a predictive tool. The *communication* and *delivering value* aspects require a more complex approach. Communication in healthcare involves multiple stakeholders who each have their own unique needs and motives. The appointment of a clinical champion can help to communicate the value of the predictive tool to the relevant stakeholders.^59^ The responsibilities of the clinical champion should centre around two key areas: adoption and promotion of the new predictive tool.^60^ For a new tool to be adopted, the clinical champion needs to disseminate the intervention to the relevant stakeholders. This includes facilitating meetings between consumers (patients), clinicians, and managerial staff of the relevant health system. The purposes of these meetings are to create an open channel for communication between stakeholders. As a result, the ideal clinical champion should be familiar with the local health system and its relevant infrastructure.^61^ The task of promotion aims to present how the new tool will benefit relevant stakeholders. This includes identifying the clinical gaps and highlighting how the tool will address these gaps. In other words, the clinical champion promotes the hidden value of the predictive tool to demonstrate its appeal in a new clinical environment. A clinical champion therefore also requires an understanding of how new tools may affect different stakeholders.^60^ It is through the clinical champion that communication can be centralized, and value delivered. Ideally, new tools should build on the shared decision‐making concept where joint decisions are made between providers and consumers of healthcare. Based on previous experience, shared decision‐making is a key strength of many predictive tools that are already used in routine clinical practice.^62^

## Integration into existing healthcare systems

All healthcare systems have pre‐existing infrastructure and patient care frameworks in place.^63^ New interventions, especially technology‐based interventions, can threaten the harmony of existing systems in play. A recent article by Liberati *et al*. suggested that although clinicians may feel they are ready to promote the integration of technology into patient care, their hospitals or healthcare systems may not be.^64^ This discourse is perhaps unsurprising given the large investments required to build hospital systems. It is therefore important to create predictive tools with the possibility of integration into existing healthcare systems. A combination of logistical and technical input is required for this to occur smoothly.

Through meetings facilitated by the clinical champion, technical requirements for predictive tool integration should be established ahead of time. This includes programming details, security measures, and data storage plans that all need consideration during the design and development of new tools. Technical experts should be available to implement these integration requirements. Some predictive tools may function well when housed on external platforms such as websites or apps. However, there is a growing shift towards formal regulatory processes for digital interventions in clinical practice. Predictive tools are considered digital medical devices in many jurisdictions and therefore may be subject to stricter regulation in the future.^65^ Upcoming predictive tools will likely require strategies around how they protect the sensitive information they capture to remain compliant with current and incoming legislation.^66^

Finally, a further consideration for integrating new tools is the available support from government or key policy makers.^67^ Using the example of the Gustilo‐Anderson classification, this was first described in 1976 as a prognostic tool to guide surgical treatment of open fractures.^68^ Two recent developments significantly increased its implementation in general clinical practice.^69^ The first occurred in 2016 with the incorporation of Gustilo‐Anderson concepts into the National Institute for Health and Care Excellence (NICE) guidelines for complex fracture management.^70^ The second and more recent development occurred in 2020, when the British Orthopaedic Association (BOA) and British Association of Plastic Reconstructive and Aesthetic Surgeons (BAPRAS) published a joint statement on the management of open fractures which used the Gustilo‐Anderson classification.^71^ This resulted in the Gustilo‐Anderson classification being used as agreed definitions for the transfer and care of all patients with open fractures, rather than limited to a specialty specific tool. This example demonstrates the significant influence that policy changes can have on predictive tool integration into existing healthcare systems.

## Continuous monitoring and evaluation

The relationships between input and output data for predictive tools may change over time. This is referred to as concept drift.^72^ As a result, all predictive tools should have a plan in place to continuously monitor and evaluate their performance.^73^ In the context of predictive tools, predicted outcomes should be recorded and analysed at scheduled intervals. This usually takes the form of follow‐up studies with updated datasets.^74^ Metrics used to assess the tool's performance, such as AUC, RMSE, sensitivity and specificity, should be updated and reported in a transparent fashion. Minor adjustments to the predictive model based on new data can improve its predictive capabilities. However, there is also a risk of invalidating the performance and supporting evidence behind a tool if substantial adjustments are made. Therefore, a clinical taskforce should be established to continuously monitor and evaluate a new predictive tool's performance systematically.

## Conclusions

This review presented seven principles that affect predictive tool implementation in clinical practice. Key areas of discussion were (1) identifying a clinical gap, (2) selecting a target user or population, (3) optimizing predictive tool performance, (4) externally validating predictive tools, (5) marketing and disseminating the tool, (6) navigating the challenges of integrating a tool into existing healthcare systems, and (7) developing an ongoing monitoring and evaluation strategy. Using these implementation principles, the development of new predictive tools can follow a structured pathway to work closely with clinical stakeholders from the outset. The end goal is to successfully implement new predictive tools into clinical practice.

## Author contributions

**Daniel Gould:** Conceptualization; formal analysis; investigation; methodology; resources; visualization; writing – original draft; writing – review and editing. **Peter Choong:** Conceptualization; methodology; supervision; writing – review and editing. **Michelle Dowsey:** Methodology; supervision; writing – review and editing. **Chris Schilling:** Conceptualization; methodology; supervision; writing – review and editing. **Yuxuan Zhou:** Conceptualization; data curation; formal analysis; investigation; methodology; project administration; resources; validation; visualization; writing – original draft; writing – review and editing.

## Funding information

Yuxuan Zhou is supported by a PhD research stipend from The University of Melbourne and HCF Research Foundation Grant.

## Conflict of interest

None declared.
